# Metal cofactor modulated folding and target recognition of HIV-1 NCp7

**DOI:** 10.1371/journal.pone.0196662

**Published:** 2018-05-01

**Authors:** Weitong Ren, Dongqing Ji, Xiulian Xu

**Affiliations:** 1 School of Physics, Nanjing University, Nanjing 210093, China; 2 School of Physical Science and Technology, Yangzhou University, Yangzhou 225002, China; Russian Academy of Medical Sciences, RUSSIAN FEDERATION

## Abstract

The HIV-1 nucleocapsid 7 (NCp7) plays crucial roles in multiple stages of HIV-1 life cycle, and its biological functions rely on the binding of zinc ions. Understanding the molecular mechanism of how the zinc ions modulate the conformational dynamics and functions of the NCp7 is essential for the drug development and HIV-1 treatment. In this work, using a structure-based coarse-grained model, we studied the effects of zinc cofactors on the folding and target RNA(SL3) recognition of the NCp7 by molecular dynamics simulations. After reproducing some key properties of the zinc binding and folding of the NCp7 observed in previous experiments, our simulations revealed several interesting features in the metal ion modulated folding and target recognition. Firstly, we showed that the zinc binding makes the folding transition states of the two zinc fingers less structured, which is in line with the Hammond effect observed typically in mutation, temperature or denaturant induced perturbations to protein structure and stability. Secondly, We showed that there exists mutual interplay between the zinc ion binding and NCp7-target recognition. Binding of zinc ions enhances the affinity between the NCp7 and the target RNA, whereas the formation of the NCp7-RNA complex reshapes the intrinsic energy landscape of the NCp7 and increases the stability and zinc affinity of the two zinc fingers. Thirdly, by characterizing the effects of salt concentrations on the target RNA recognition, we showed that the NCp7 achieves optimal balance between the affinity and binding kinetics near the physiologically relevant salt concentrations. In addition, the effects of zinc binding on the inter-domain conformational flexibility and folding cooperativity of the NCp7 were also discussed.

## Introduction

The nucleocapsid protein(NCp7) of human immunodeficiency virus 1(HIV-1) is a small zinc-binding protein derived from the cleavage and processing of the HIV structural protein Gag [1–4]. It plays critical role in the HIV-1 replication and facilitates numerous processes in the viral life cycle. For example, by interacting with a 120-nucleotide region of the unspliced viral RNA, named as the *Ψ*-site, the NCp7 enables the encapsulation of viral genome into new mature virions [5]. It enhances the reverse transcription via its nucleic acid chaperone activity which induces the structural rearrangement of nucleic acids into the most stable conformation [4, 6–9].

**Fig 1:**
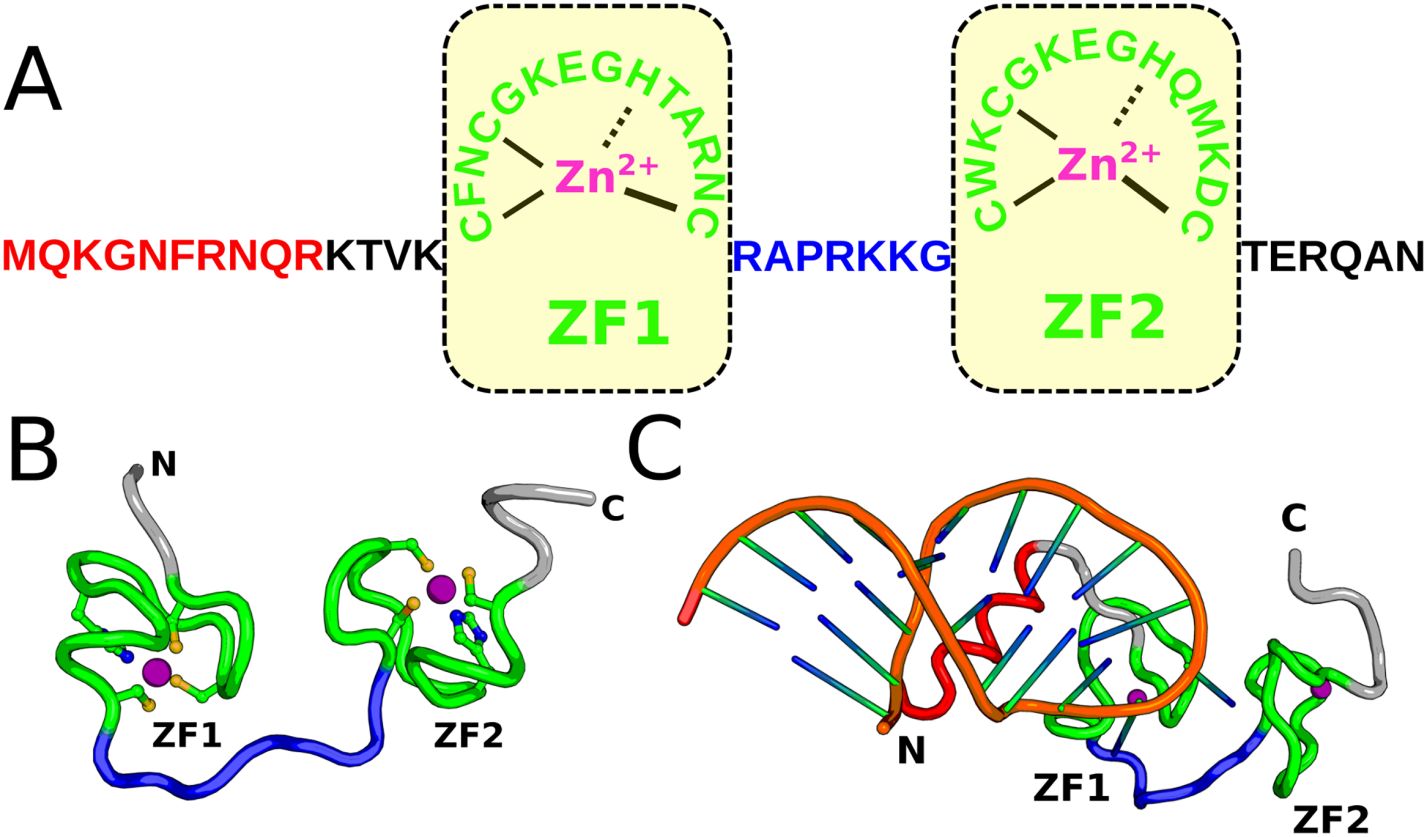
(**A**) Sequence and structural features of NCp7. The two CCHC-type zinc finger motifs are highlighted by yellow rectangles. (**B**, **C**) The three-dimensional structure of zinc-chelated NCp7(PDB: 1esk) and NCp7-SL3 complex(PDB: 1a1t) respectively. Color scheme: green, ZF1 and ZF2; purple, zinc ions; blue, linker; red, N-terminal helix; orange, SL3.

Structurally, the NCp7 is a 55-residue polypeptide and consists of two highly conserved CCHC-type zinc finger motifs connected by a short linker as showed in Fig 1. Both the proximal (ZF1) and distal (ZF2) zinc fingers have the sequence pattern of Cys-X2-Cys-X4-His-X4-Cys (the X is any substituted amino acid), with Zn^2+^ coordinating with three cysteines and one histidine [10, 11]. Experimental data showed that without zinc binding, the NCp7 behaviors as an intrinsically disordered protein [12–17]. Upon zinc binding, the two zinc-finger domains get folded to nearly identical structures. Folding of the NCp7 forms a hydrophobic plateau [18, 19], which plays a key role in the recognition of nucleic acids.

Despite of the fact that the two zinc finger domains adopt quite similar folded conformations, the ZF1 shows higher zinc affinity and stability compared to the ZF2, as demonstrated by Mely and coworkers using fluorescence spectroscopy [15]. In addition, the two zinc fingers contribute differently to the activity of the NCp7. The ZF1 plays more important role in the RNA helix reconfiguration, which is essential for the reverse transcription [20]. Furthermore, it has been shown that the ZF1 with flanking basic segments represent the minimal region of the NCp7 for targeting chaperone function [21]. Very recently, Deshmukh and coworkers showed that the ZF2 populates some excited states transiently and sparsely [22]. These “dark” states, which account for around 1.0% of the total population, are featured by the disrupture of one Cys-Zn or His-Zn coordination bond. Computational work also identified the distinctions in the protein packing and electrostatic screening between the two zinc-finger domains [23]. NMR and mass spectroscopy studies showed that ZF2 is more reactive towards zinc-ejecting small molecules [24].

Using solution NMR and X-ray scattering techniques, Deshmuch et al. quantitatively characterized the conformational space sampled by NCp7 [25]. They found that the solution dynamics of NCp7 are featured by transitions between compact conformations with weak inter-domain interactions and more extended conformations without inter-domain interactions. Such inherent conformational plasticity and flexibility enables adaptive binding of NCp7 to different target nucleic acids, and is essential to the functional versatility of NCp7 [25–27].

The vital role of the zinc binding on the biological functions of the NCp7 has been well demonstrated by large number of experimental studies. Zinc ejection or single-point mutations suppressing the zinc affinity cause complete loss of virus infectivity [28–32]. These disrupted or mutated NCp7 proteins fail to recognize and pack the genomic RNA into new virus particles. Even subtle alterations of the native zinc finger structures tend to reduce the nucleic acid chaperone activity of NCp7 dramatically [33, 34].

Considering the crucial role of the NCp7 on the virus replications, it has long been taken as a potential therapeutic target of HIV-1 by disrupting the zinc binding ability [1, 29, 35, 36]. Apparently, understanding the molecular mechanism of how the zinc ions modulate the conformational dynamics and functions of the NCp7 are essential for the drug development and HIV-1 treatment. Computationally, although all-atom detailed molecular dynamics (MD) can be used to characterize the role of zinc binding on the structure and dynamics of the NCp7, the accessible timescales are typically limited to microsecond [37–39], which are much shorter compared to the biological timescales for the NCp7 folding and target recognition.

Here, we used a coarse-grained model with structure-based potential [40, 41] and implicit consideration of ligand binding to investigate the zinc ion coupled folding and target RNA binding of the NCp7. Our results showed that the binding of zinc ions tends to modulate the folding pathways of the two zinc fingers and make the transition state ensemble less structured. In addition, we revealed the mutual coupling between the zinc ion binding and the target RNA recognition, in which the formation of the NCp7-RNA complex reshapes the intrinsic energy landscape of the NCp7 and increases the stability and zinc affinity of the two zinc fingers. The important role of electrostatic interactions in the association of NCp7 to nucleic acid was also demonstrated. These results provide insights into the crucial role of the zinc cofactors on the structure and target RNA recognition of the NCp7 during the HIV-1 life cycle.

## Materials and methods

In this work, we used a coarse-grained model for the protein and RNA molecules. Each residue of the NCp7 was represented by a spherical bead locating at the C_*α*_ atom. The intra-molecule interactions were described by a structure-based energy function [41]. In addition, we considered the sequence dependence of the native interactions and the secondary structure propensity of the protein sequence [42, 43]. The structure-based energy function was given by [41–43]

$$\begin{array}{c} \begin{array}{cc} V(R|R_{0}) & =V_{b}+V_{flp}+V_{loc}+V_{nloc}+V_{exv}\\ & =\sum_{i}K_{b}{\left(r_{i}-r_{i}^{0}\right)}^{2}+\sum_{i}V_{a}\left(\theta_{i}\right)+\sum_{i}V_{dih}\left(\phi_{i}\right)\\ & +\lambda \sum_{j=i+2}\epsilon_{ij}^{1,3}exp(-\frac{{\left(r_{ij}-r_{ij}^{0}\right)}^{2}}{2\omega^{2}})\\ & +\lambda \sum_{j=i+3}\epsilon_{ij}^{1,4}exp(-\frac{{\left(\phi_{ij}-\phi_{ij}^{0}\right)}^{2}}{2\omega_{\phi }^{2}})\\ & +\lambda \sum_{j>i+3}^{native}\epsilon_{ij}^{nloc}[5{\left(\frac{r_{ij}^{0}}{r_{ij}}\right)}^{12}-6{\left(\frac{r_{ij}^{0}}{r_{ij}}\right)}^{10}]\\ & +\sum_{j>i+3}^{nonnative}\epsilon {\left(\frac{C}{r_{ij}}\right)}^{12} \end{array} \end{array}$$ (1)

The term *V*_*b*_ considers the covalent connectivities of the peptide chain and was represented by harmonic potential. The *V*_*flp*_ describes the sequence dependent chain flexibility and secondary structure propensity, and was constructed based on statistical survey of the local structures of the loop segments in protein data bank [44]. The *V*_*loc*_ and *V*_*nloc*_ represents the structure-based potentials of the local and non-local contacts, with the relative weights of the pairwise interactions being derived according to all-atom force field. The parameter λ controls the overall stability of the protein, and was set as 0.7 in this work. The details of the energy function can be found in Ref. [42, 43, 45].

The effect of Zn^2+^ binding was modelled by the implicit ligand-binding model [46]. In this model, the Zn^2+^ ions were not included explicitly. Instead, the energetic effect of Zn^2+^ binding was modelled by strengthening the contacting interactions formed by the liganding residues of Zn^2+^ at the native state. In this work, the Zn^2+^ binding site of each zinc-finger domain given in the Protein Data Bank file (PDB code: 1esk) was used. The zinc-binding mediated contacts have the interactions given by

$$\begin{array}{c} V_{bind}=\sum_{\begin{array}{c} ligand-mediated\\ contact-pairs \end{array}}-C\cdot exp\left(-\frac{{\left(r_{ij}-r_{ij}^{0}\right)}^{2}}{2\sigma^{2}}\right) \end{array}$$ (2)

where *C* was set as 1.5 kcal/mol, with which the NCp7 stays well folded at saturated zinc concentrations. *σ* represents the width of the Gaussian potential for the ligand-mediated contacts and was set as 0.15Å. $r_{ij}^{0}$ is the distance between the liganding residues in the native conformation. The transitions between the Zn^2+^-bound and unbound states were realized by standard Metropolis Monte Carlo simulations. Specifically, Zn^2+^ binding is assumed to be diffusion limited and occurs with the rate *k*_*on*_[*Zn*^2+^], where *k*_*on*_ is the second-order rate constant, and [*Zn*^2+^] is the zinc concentration. The Zn^2+^ dissociation rate is given by $k_{u}^{0}exp\left(-V_{bind}/k_{B}T\right)$, where $k_{u}^{0}$ is the intrinsic off rate taken from Ref. [46], *V*_*bind*_ is the binding energy that depends on the conformations of the binding site, and *k*_*B*_*T* is the thermal energy. In the simulations, *k*_*on*_[*Zn*^2+^] as a whole was controlled by an input parameter.

The RNA model developed in Ref. [47] was used for the intra-molecule interactions of the RNA molecule. In this model, each nucleotide was coarse-grained as three beads, i.e., phosphate(P), sugar(S), and base(B). Bases were classified as two types, i.e., purine(R) and pyrimidine(Y). The energy function of the RNA molecule was given by

$$\begin{array}{c} \begin{array}{c} V_{RNA}\left(R|R_{0}\right)=V_{local}+V_{contact}+V_{ele}+V_{exv} \end{array} \end{array}$$ (3)

The local term *V*_*local*_ includes the potentials of the virtual bond, virtual angles and virtual dihedrals formed by covalently connected beads. Only the virtual angles formed by *B*(*i*) − *S*(*i*) − *P*(*i* + 1), and the virtual dihedral angles by *B*(*i*) − *S*(*i*) − *P*(*i* + 1) − *S*(*i* + 1) were considered, where *i* is the residue number. The energy function of the local term was given by

$$\begin{array}{c} \begin{array}{cc} V_{local} & =\sum_{\eta (ibd)}K_{b}^{\eta }{\left(r_{ibd}-r_{ibd}^{0}\right)}^{2}+\sum_{\eta (iba)}K_{\theta }^{\eta }{\left(\theta_{iba}-\theta_{iba}^{0}\right)}^{2}\\ & +\sum_{\eta (idih)}\{K_{\phi }^{\eta }\left(1-cos\left(\phi_{idih}-\phi_{idih}^{0}\right)\right)\\ & +\frac{1}{2}K_{\phi }^{\eta }\left(1-cos3\left(\phi_{idih}-\phi_{idih}^{0}\right)\right)\} \end{array} \end{array}$$ (4)

The *V*_*contact*_ represents the LJ type interactions for the native contacts.

$$\begin{array}{c} V_{contact}=\sum_{i,j\in S,B}^{native}\epsilon_{\xi }[5{\left(\frac{r_{ij}^{0}}{r_{ij}}\right)}^{12}-6{\left(\frac{r_{ij}^{0}}{r_{ij}}\right)}^{10}] \end{array}$$ (5)

The term *V*_*exv*_ considers the excluded volume effect.

$$\begin{array}{c} V_{exv}=\sum_{ij,nonlocal}^{nonnative}\epsilon_{ex}{\left(\frac{d}{r_{ij}}\right)}^{12} \end{array}$$ (6)

The *V*_*ele*_ is the electrostatic potential between the charged beads and takes Debye-H*ü*ckle form

$$\begin{array}{c} V_{ele}=\sum_{i,j\in charge}\frac{1}{4\pi \epsilon_{0}\epsilon_{r}}\frac{q_{i}q_{j}}{r_{ij}^{2}}exp(-\frac{r_{ij}}{\lambda_{D}}) \end{array}$$ (7)

where *q*_*i*_ and *q*_*j*_ are the charges of the residue *i* and *j* respectively. *ϵ*_0_ and *ϵ*_*r*_ are the dielectric constant and the relative dielectric constant for water. λ_*D*_ is the Debye length and is represented as:

$$\begin{array}{c} \lambda_{D}=\sqrt{\frac{\epsilon_{0}k_{B}T}{4e^{2}I}} \end{array}$$ (8)

where *e* is the elementary electric charge. *I* is ion strength and is dependent on the salt concentration.

The interactions between the NCp7 and the RNA molecule include two terms, the structure-based LJ type interactions (based on the structure with PDB code: 1a1t), which are applied to the contacting pairs formed in the native structure of the complex, and the electrostatic interactions, which are applied to the charged beads. In protein residues, the charges of the Glu, Asp, Arg and Lys were set as −1.0, −1.0, +1.0 and +1.0, respectively. The phosphate beads of the RNA have the charge of −1.0. The relative weights of the contacting interactions between NCp7 and RNA molecule are proportional to the values obtained from the all-atom force field by using energy decomposition [48, 49].

All the simulations were conducted by Langevin dynamics using the CafeMol3.0 software package at the temperature of 300 K and salt concentration of 200 mM [50]. The relative dielectric constant was set as *ϵ*_*r*_ = 80.0.

## Results

### Zinc binding and protein stability

**Fig 2:**
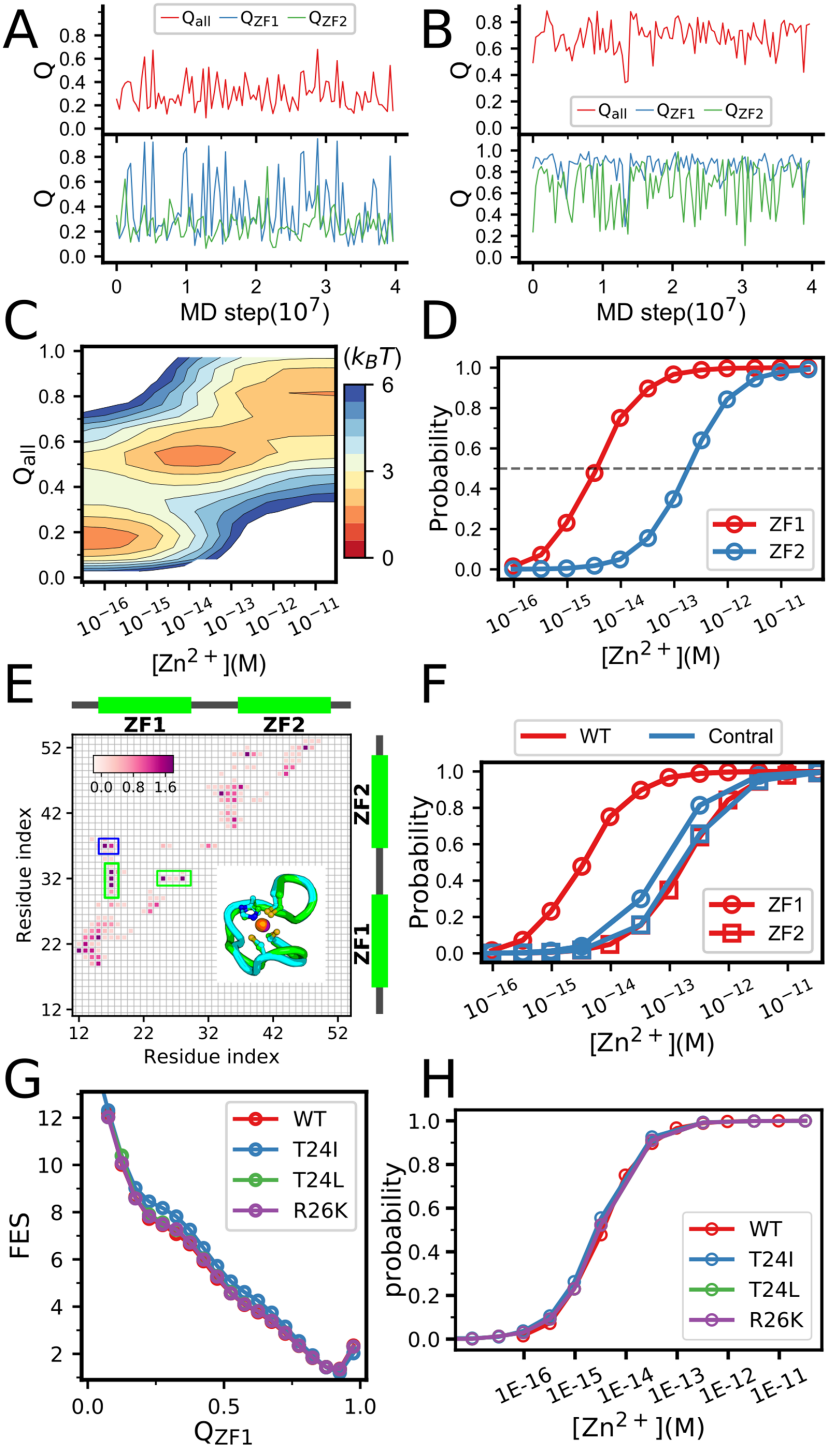
(**A**, **B**) Representative folding/unfolding trajectories of the NCp7 without zinc binding (**A**) and with saturated Zn^2+^ concentration (**B**). (**C**) The free energy profiles of NCp7 along the *Q* value of the whole protein (*Q*_*all*_) and Zn^2+^ concentrations. (**D**) Binding probabilities of the zinc ions in the ZF1 (red) and ZF2 (blue) as a function of Zn^2+^ concentrations. The concentration leading to binding probability of 0.5 gives the dissociation constant. By comparing the calculated Zn^2+^-binding affinity of ZF1 with that measured in experiments, we can approximately map the input binding rate to the zinc concentrations. (**E**) The strengths of the contacting interactions in the NCp7 obtained by energy decomposition with all-atom force field [51, 52]. The contacts within blue box correspond to the inter-domain interactions. Those within green boxes correspond to the ZF1-linker interacting contacts. The structural alignment between ZF1(green) and ZF2(blue) is showed in the inset. (**F**) Probabilities of zinc binding as a function of zinc concentrations for the ZF1 (circle) and ZF2 (square) from the control simulations (Control, blue). For comparison, the results of the simulations without modifying the interactions are also shown (WT, red). In the control simulations, the ZF1-linker interactions were removed and the contacting interactions in the ZF1 and ZF2 have identical strengths. (**G**) Comparison of the free energy profiles for wild type ZF1 and single-point mutated ZF1 species(T24I, T24L and R26K) at saturated zinc concentration. Here *Q*_*ZF*1_ is the fraction of formed native contacts at ZN1. (**H**) Probabilities of zinc binding as a function of zinc concentrations for wild type ZF1 and single-point mutations of ZF1.

Previous experimental works showed that the NCp7 is mostly unstructured without zinc binding. In presence of zinc ions, the protein gets well folded. In Fig 2A and 2B, we plotted the fraction of the formed native contacts (*Q* value) for the whole NCp7 (top) and the two component zinc-finger domains (bottom) without zinc binding (A) and with high (saturated) zinc ion concentration (B). One can see that without zinc biding, the *Q* value of the sampled structures are mostly less than 0.4, whereas at high zinc ion concentrations, the *Q* values are dominantly larger than 0.7, suggesting that with the current parameter set, the experimentally observed effects of zinc binding on the protein stability can be well reproduced. Interestingly, although the same parameter set was used for the two zinc-finger domains, the resulted stabilities and zinc-binding affinities are largely distinct. At a given zinc concentration, the ZF1 is much more stable than the ZF2. In addition, the *K*_*d*_ value of the zinc binding for the ZF1 is significantly smaller (the affinity is higher) than that of the ZF2 (∼20.0 fM) as shown in Fig 2D. Consequently, the folding of the NCp7 may involve an intermediate, in which the ZF1 is well folded, whereas the ZF2 is still unstructured as shown in the two-dimensional free energy landscape (Fig 2C). Our simulation results are consistent with previous experimental data, which showed that the ZF1 has higher stability and zinc affinity than the ZF2 (with dissociation constants of ∼3.6 fM and ∼20.6 fM, respectively) [15].

We note that the difference of the binding affinity between the ZF1 and F2 is larger in the simulation data than that in the experimental data. Possible reason is that the current ligand binding model is a structure-based model, in which non-native interactions between the ligand and the protein were ignored and only the native interactions observed in the native structure were included. Such an approximation may introduce biases to the simulated binding affinity.

More closed investigations to structure and interaction details showed that the ZF1 has some more strongly interacted native contacts, especially at the N-terminal part, compared to the ZF2 (Fig 2E), although the two zinc-finger domains have nearly identical three-dimensional structures (Fig 2E). In addition, the Asn17 in the ZF1 strongly interacts with the Pro31/Arg32 of the linker, whereas the interaction between the ZF2 and the linker are much weaker (Fig 2E). Such differences may contribute to the different stabilities of the two domains. As a control, we also conducted simulations with the contacting interactions for each pair of the contacting residues of the protein equally weighted. Meanwhile, we removed the contacting interactions between the Asn17 of ZF1 and the Pro31/Arg32 in the linker. The resulted zinc binding affinities of the two fingers become comparable in the control simulations(Fig 2F), supporting the above argument about the key factors resulting in the different behaviors of the two finger domains. These results are consistent with the previous findings that the non-symmetrical contacts of the linker residues with the two zinc fingers contribute to the different dynamics of ZF1 and ZF2 [27].

As the NCp7 shows prominent sequence variations particularly in the ZF1, which could affect the protein folding and zinc coordination. To investigate the effect of such sequence variations on NCp7 folding and zinc binding. we performed additional simulations for three mutants, which introduce the mutation T24I, T24L, and R26K, respectively. As shown in Fig 2G and 2H, all these single-point alterations on the protein sequence have little effect on the ZF1 folding and zinc binding.

### Zinc binding modulated inter-domain conformational fluctuations

**Fig 3:**
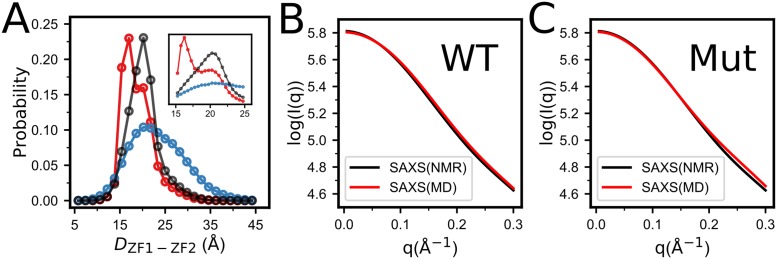
(**A**) Distributions of the distances between the centers of mass of the ZF1 and ZF2 without zinc biding (blue), and with intermediate (black) and saturated Zn^2+^ (red) concentrations. The inset shows the locally enlarged plot. (**B**, **C**) The SAXS profiles computed from the structures sampled by simulations of “wild type” protein (**B**, WT) and the “mutant” with the inter-domain contacts. removed (**C**, Mut). The SAXS profile calculated based on the NMR structural ensemble is also shown for comparison.

Earlier studies showed that the two zinc-finger domains of the NCp7 behave as independently folded, non-interacting domains connected by a flexible linker [13]. Subsequent studies indicated the presence of weak and transitory inter-domain interactions [15, 16]. Consequently, the solution behavior of NCp7 can be considered as a rapid equilibrium between weakly interacting and non-interacting inter-domain conformations [25, 26]. To characterize the inter-domain conformational fluctuations, we calculated the distributions of the inter-domain distances *D*_ZF1−ZF2_, which is represented by the distance between the centers of mass of the two zinc-finger domains, in a wide range of Zn^2+^ concentrations. As showed in Fig 3A, without zinc binding, both the ZF1 and ZF2 are disordered, leading to a wide distribution of *D*_ZF1−ZF2_ with one peak. Increasing the Zn^2+^ concentration stabilizes the conformations of the two zinc-finger domains, which leads to shifts of the peak position to the lower values of the *D*_ZF1−ZF2_ and narrowing of the distribution. When Zn^2+^ concentration approaching saturation, two different peaks show up, demonstrating the heterogeneity of the inter-domain conformational distributions. Since the two zinc-finger domains are well folded with saturated zinc concentrations, the observation of two peaks of the distance distributions is resulted from two different inter-domain conformations. The peak with small inter-domain distance corresponds to the conformations with well formed inter-domain contacts. Whereas for the wide peak with larger inter-domain distance, the contacts are transiently broken, leading to increased inter-domain separations and fluctuations. Our results are consistent with previous experimental observations and demonstrated that the zinc binding is able to modulate the inter-domain conformations. Such inherent conformational flexibility may be functionally important, enabling adaptive binding of the NCp7 to different recognition elements [53, 54].

The important role of the inter-domain contacts in the global conformational fluctuation of the NCp7 can also be demonstrated by Fig 3B and 3C, in which we showed the SAXS profiles calculated based on the sampled snapshots with (B) and without (C) the inter-domain contacting interactions at saturated zinc concentration. The Fast-SAXS-pro method was used to compute the SAXS profiles of NCp7 from the sampled coarse-grained conformations [55]. The SAXS profiles calculated based on the structure ensemble by solution NMR (PDB code: 5i1r) are also shown for comparison [25]. One can see that the SAXS profile from the simulations with inter-domain contacting interactions can well reproduce the results from the NMR structural ensemble. In comparison, the profile from the sampled structural ensemble without considering the inter-domain contacting interactions shows minor, but significant, deviations to that of the NMR structures.

**Fig 4:**
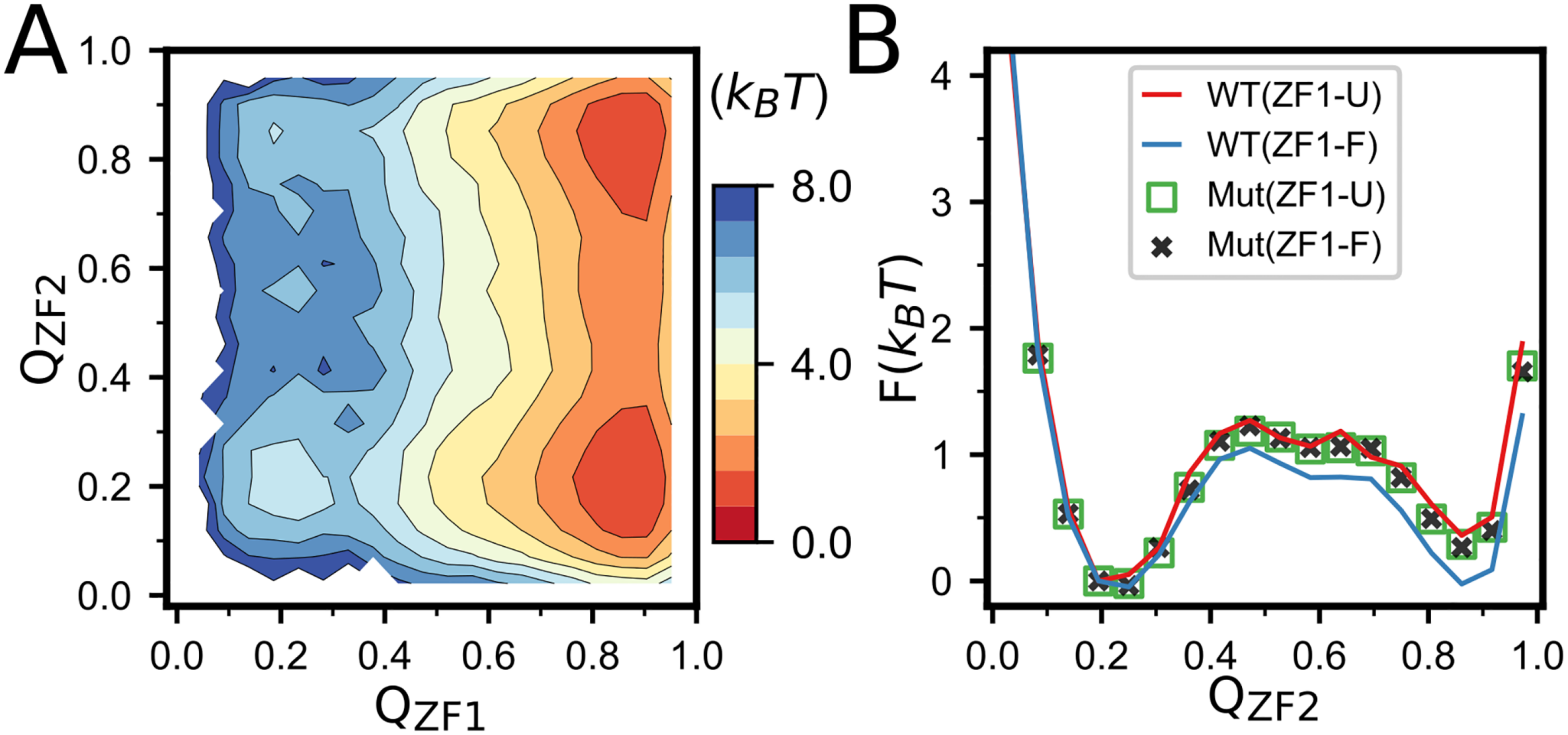
(**A**) Free energy surface of NCp7 projected on *Q*_*ZF*1_ and *Q*_*ZF*2_ at intermediate concentration of Zn^2+^. (**B**) One-dimensional free energy profiles projected onto the *Q* value of the ZF2 with the ZF1 well-folded (red) and unstructured (blue), respectively at intermediate zinc concentration. For comparison, the corresponding free energy profiles from the simulations. ‘U’ and ‘F’ denote the unfold and fold states of the ZF1, respectively. with the inter-domain interactions removed are also shown (dots).

The inter-domain contacts may introduce coupling between the folding of the two domains. As shown in the two-dimensional free energy profile projected onto the *Q* values of the two zinc-finger domains (Fig 4A) and the projected one-dimensional free energy profile (Fig 4B), the folded conformations of the ZF2 are slightly more stable when the ZF1 stays at folded conformation(Fig 4B), suggesting weak folding cooperativity between the two domains. In comparison, for the control simulations with the inter-domain contacting interactions removed, the ZF1 conformation has no effects on the stability of the ZF2 (Fig 4B), suggesting that the cooperativity of the folding of the two domains is mediated by the inter-domain contacting interactions.

### Effect of zinc binding on the folding dynamics of zinc-finger domains

**Fig 5:**
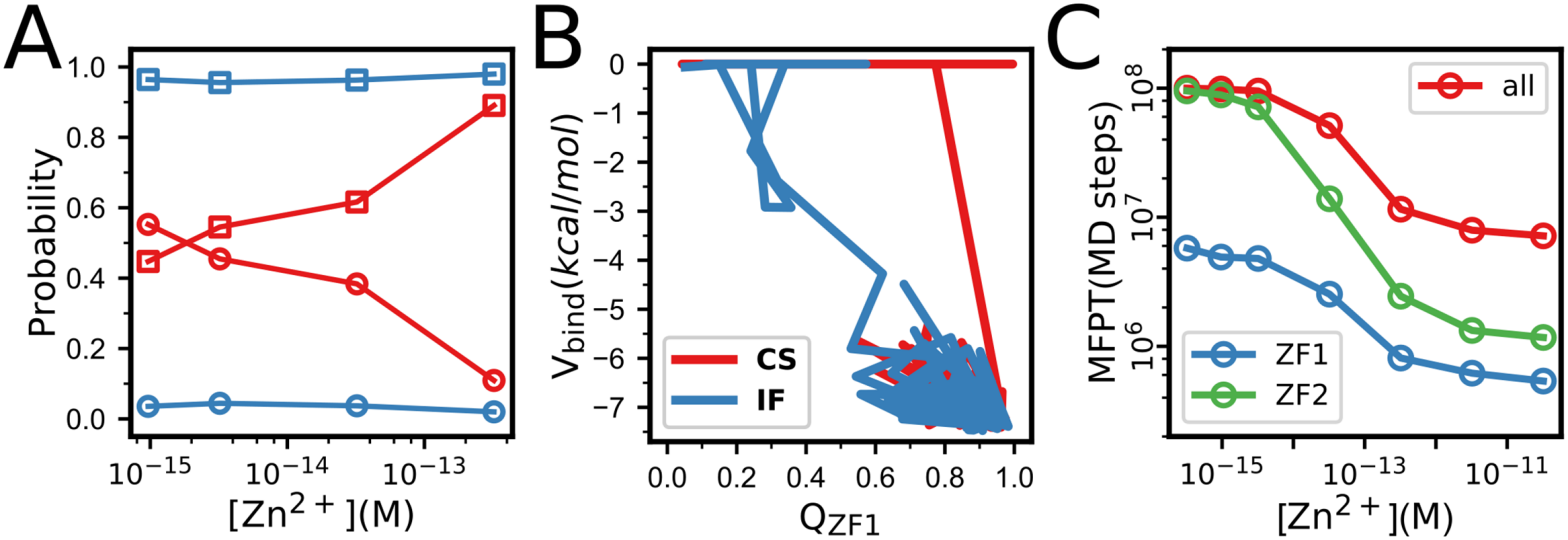
(**A**) Probabilities of the two different folding mechanisms of ZF1(red) and ZF2(blue) at different Zn^2+^ concentrations. The squares represent the “binding induced folding” pathway, whereas the circles represent the “conformational selection” pathway. (**B**) Represent folding trajectories of ZF1 with “binding induced folding”mechanism (blue) and “conformational selection” mechanism (red), projected onto the space formed by the binding energy and *Q* value. (**C**) The mean first passage time(MFPT) of the folding of NCp7(red), ZF1(blue) and ZF2(green) as a function of Zn^2+^ concentrations.

The NCp7 has also been used as a model protein to study the role of metal cofactors in the protein folding [56–59]. Conceptually, the two zinc-finger domains can fold with two possible mechanisms: i) the zinc ions bind at the unfolded state and direct/modulate the whole folding processes, which is often called as “binding induced folding” [38, 60, 61]; and ii) the peptide can fold spontaneously, and the zinc ions bind to and stabilize the folded structure, which can be termed as “conformational selection” mechanism. Our simulation results show that for the ZF1, both mechanisms can coexist as shown in the Fig 5A and 5B. The relative importance of the two mechanisms depends on the zinc concentrations. At low zinc concentrations, the “conformational selection” mechanism has higher probability. Whereas at saturated zinc concentration, the “binding induced folding” mechanism becomes more probable. In comparison, for the ZF2, the folding dominantly follows the “binding induced folding” even at very low zinc concentrations. Such differences of the zinc-coupled folding mechanisms arises from the large difference of the folding rates of the two zinc-finger domains. The folding rate of the ZF1 is much higher than that of the ZF2, as shown in Fig 5C. Consequently, the ZF1 has chance to get folded before the binding of the zinc ions. In comparison, due to the slow folding rate, the zinc ions tend to bind with the ZF2 before the folding occurs, leading to the dominance of the “zinc binding induced folding” mechanism. It is worth noting that the occurrence of the folding events with the above two mechanisms may depend on the value of the parameter λ in Eq 1. However, the relative importance of the two folding mechanisms in the ZF1 and ZF2 is less sensitive to the parameter λ, since the same value is used for the two zinc-finger domains.

**Fig 6:**
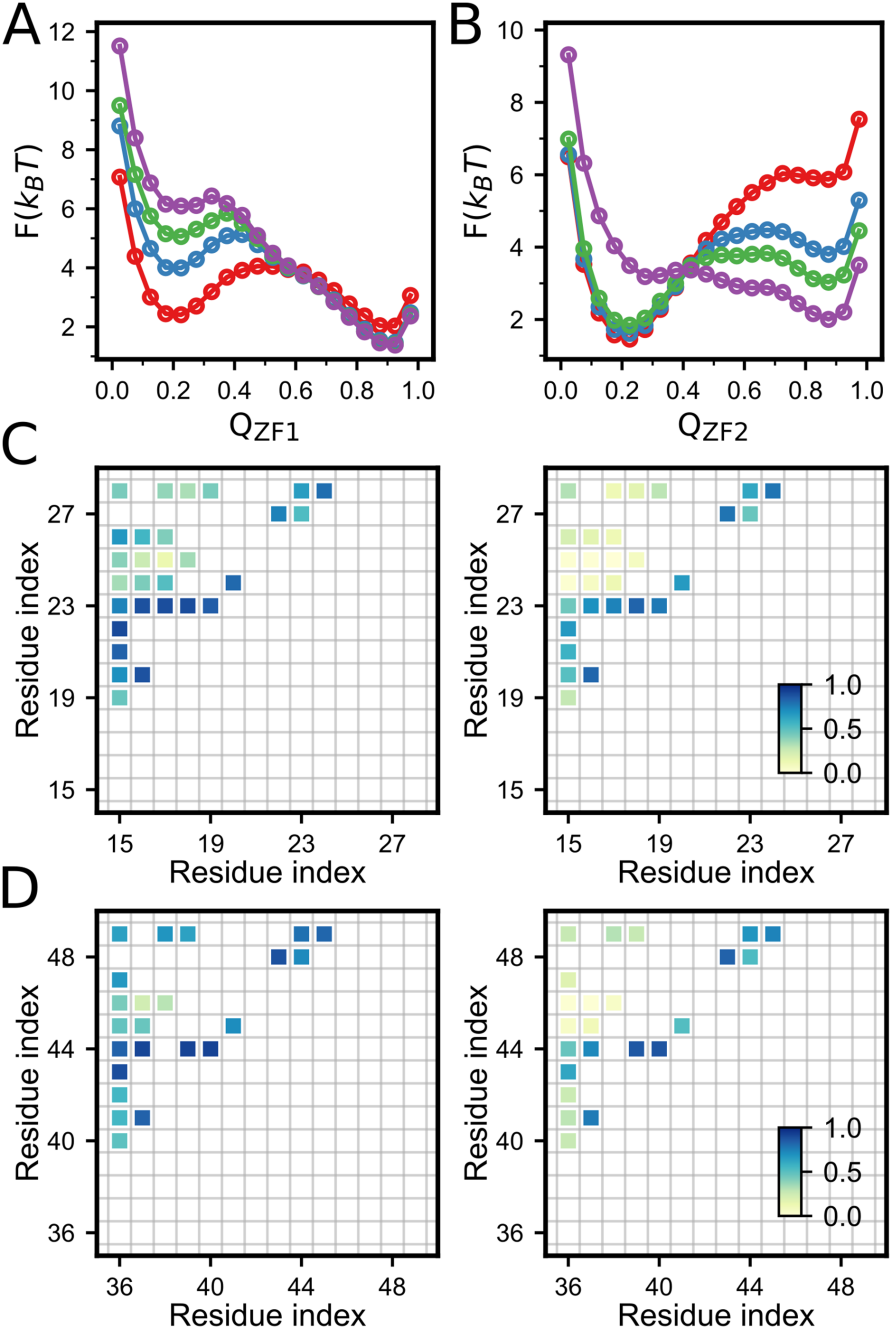
(**A**, **B**) Free energy profiles of ZF1(A) and ZF2(B) with different zinc concentrations. From red to purple, the zinc concentration increases gradually. (**C**, **D**) The formation probabilities of the native contacts at the transition state ensemble of ZF1(**C**) and ZF2(**D**) without zinc binding (left) and with saturated zinc concentration (right), respectively.

The role of zinc binding on the folding of the two zinc-finger domains can also be illustrated by the free energy profiles projected onto the *Q* values. Fig 6A and 6B shows the free energy profiles of the ZF1 (A) and ZF2 (B) at different zinc concentrations. We can see that for both zinc-finger domains, the positions of the free energy barriers shift towards the unfolded state with the increasing of zinc concentrations, suggesting that the zinc binding makes the transition state ensemble less structured. Similar results can be found in the contact probabilities of the transition state ensemble calculated based on the snapshots sampled from the narrow region around the free energy barrier, which show that native contacts are formed with higher probability at low zinc concentrations than those at at saturated zinc concentrations (Fig 6C and 6D). Since zinc binding always increases the protein stability, the effect of zinc binding on the position of the transition state is in accordance with the Hammond effect typically observed in the mutation, temperature and denaturant induced protein folding/unfolding transitions [62, 63].

### Effects of zinc binding on the target RNA recognition of NCp7

The biological functions of the NCp7 in the replication of HIV-1 rely on the proper recognition to the targets. Mutations suppressing Zn^2+^ binding ability lead to uninfectious virions. During the virus assembly, the NCp7 needs to bind with a 120-nucleotide RNA, which is known as *Ψ*-packaging signal and contains four stem-loops (SL1-SL4) [5, 31]. Among the four stem-loops, the SL2 and SL3 are critical to the viral genome recognition and specific packaging of the unspliced genome during virus assembly.

**Fig 7:**
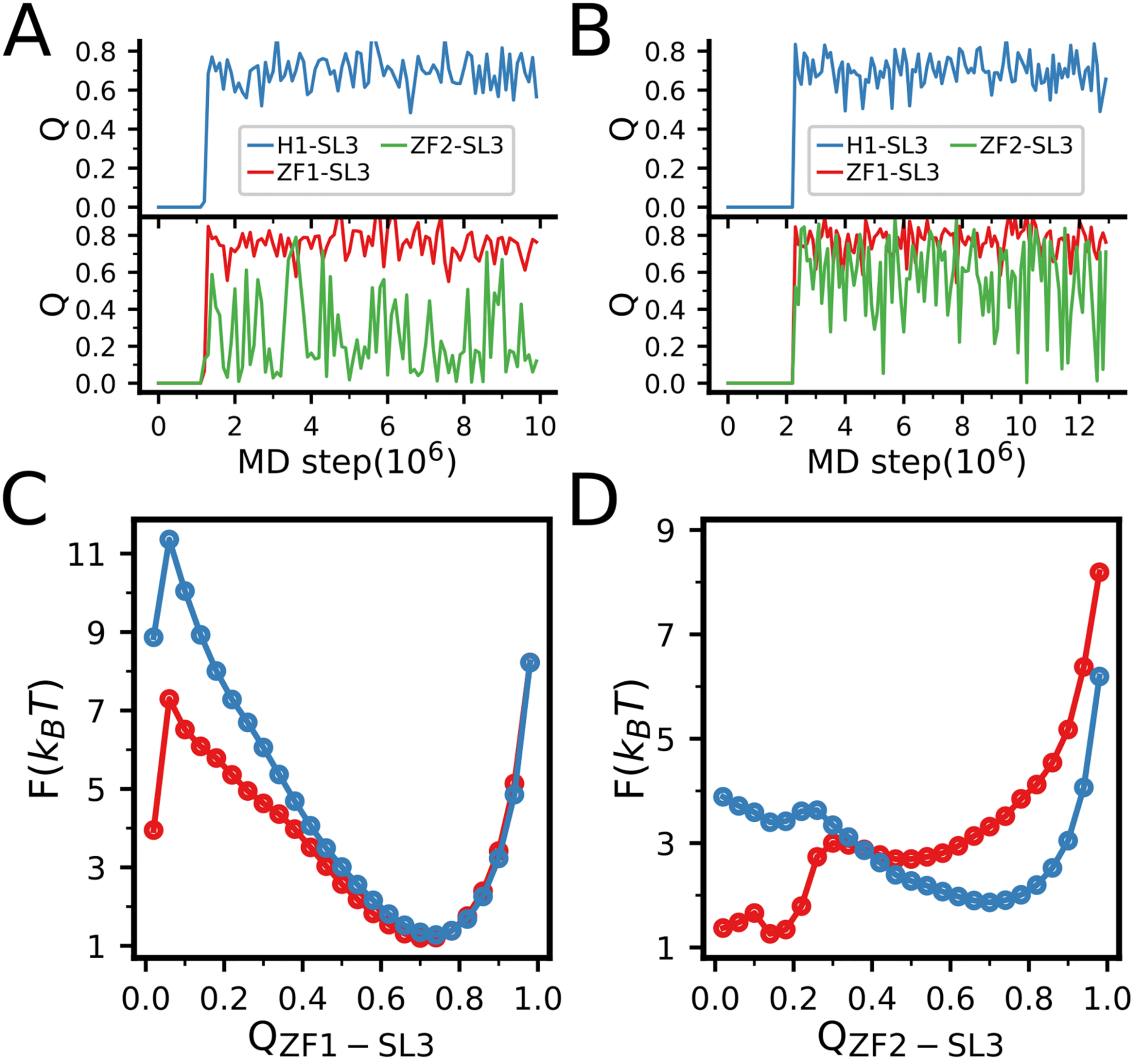
(**A**, **B**) Representative trajectories of the NCp7 binding to the SL3 RNA without zinc binding(**A**) and with saturated Zn^2+^ concentration (**B**), as monitored by the inter-chain *Q* values. (**C**, **D**) The free energy profiles of the ZF1(**C**) and ZF2(**D**) binding to SL3 without zinc binding (red) and with saturated Zn^2+^ concentration extracted by using umbrella sampling and re-weighting.

Here, by performing MD simulations on the NCp7-SL3 complexes, we studied the effects of zinc binding on the association of NCp7 to SL3. Firstly, we conducted extensive MD simulations on the association of NCp7 to SL3 RNA. As shown in previous work [18], in addition to the two zinc fingers, the N-terminal helix can also contribute to the NCp7-RNA interactions (Fig 1C, red). Therefore, in the simulations of the NCp7-SL3 binding, we included the N-terminal helix (H1). Starting from the initial structures with the NCp7 and SL3 well separated (The centers of mass were separated by 50.0 Å), we simulated the binding and subsequent relaxation processes. Fig 7A shows three inter-chain *Q* values, i.e., *Q*_H1−SL3_, *Q*_ZF1−SL3_, and *Q*_ZF2−SL3_. Here *Q*_H1−SL3_ (*Q*_ZF1−SL3_, *Q*_ZF2−SL3_) is the fraction of formed native contacts between the H1 (ZF1, ZF2) and SL3. One can see that, for both the H1 and ZF1, once binding to the SL3, they tend to stay stably at the bound state even without zinc binding. In comparison, the ZF2 hops between the bound state and unbound state rapidly. Zinc binding increases the dwelling time of the bound state.

Due to the high affinity between the ZF1 and SL3, it is difficult to directly sample the reversible binding/unbinding transitions in the MD simulations. To more quantitatively characterize the effect of zinc binding on the recognition between the two zinc fingers and the SL3, we conducted umbrella sampling with the distance between the centers of mass of the residue groups from the ZF1 (Phe16, Asn17, Ile24 and Ala25) and the SL3 (A211 and G212) being the reaction coordinate. Harmonic potentials were used to constrain the distances to the reference values ranging from 6.2 to 22.2Å, with 10 evenly sized windows, which correspond to relatively large protein concentrations. The strength of the harmonic potential was set to 1.0 kcal/mol. Under each window, 10 independent trajectories were collected. The simulations were conducted without zinc binding and with saturated Zn^2+^ concentration. By re-weighting the sampled structures using MBAR [64], we can construct the free energy profiles along different reaction coordinates. Fig 7C and 7D show the free energy profiles along the reaction coordinates *Q*_ZF1−SL3_ and *Q*_ZF2−SL3_.

In the umbrella sampling, we have not observed unbinding event of H1 once binding to SL3. This tight binding of H1 to SL3 may be attributed to the more condensed contacting interactions between the H1 and the major groove of the SL3 in the NCp7-SL3 complex. In comparison, zinc binding has significant effects on the binding free energy profiles of the ZF1 and ZF2. Particularly, the zinc binding contributes to the ZF2-SL3 affinity by ∼3.0 kcal/mol, which makes the otherwise unfavorable binding at apo condition much more favorable after zinc binding. Previous experimental work suggested that the ZF2 is responsible for the binding specificity in the NCp7-SL3 recognition [1, 27]. The strong dependence of the ZF2-SL3 affinities on the zinc binding suggests the crucial role of zinc ions on the specific target RNA recognition of the NCp7.

**Fig 8:**
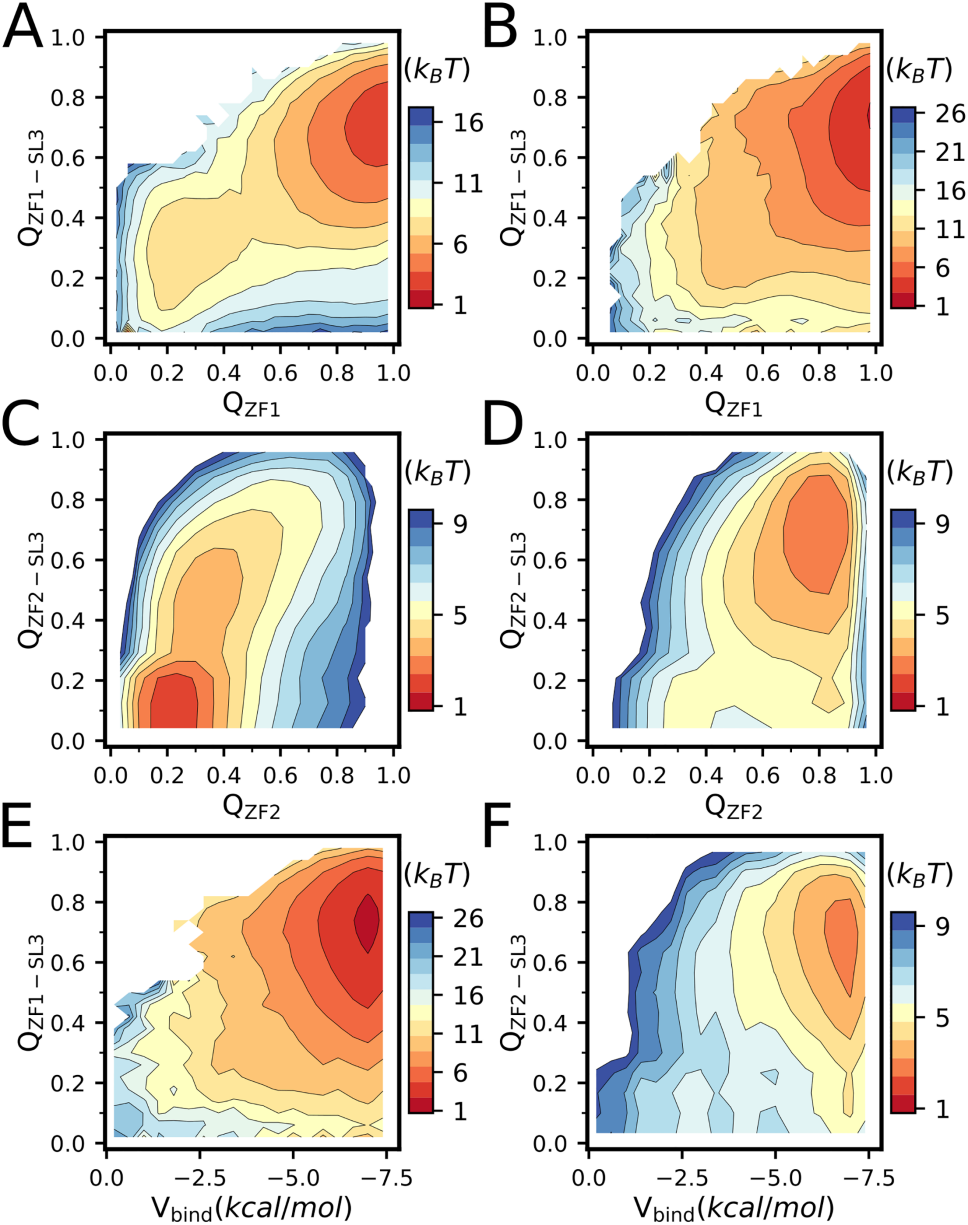
(**A**, **B**) Free energy profiles by umbrella sampling projected onto the conformational space formed by *Q*_ZF1−SL3_ and *Q*_*ZF*1_ without Zn^2+^(**A**) and with saturated zinc concentration (**B**). (**C**, **D**) Same as (**A**, **B**) but projected onto the conformational space formed by *Q*_ZF2−SL3_ and *Q*_*ZF*2_. (**E**) Free energy profiles projected onto the conformational space formed by *Q*_ZF1−SL3_ and the zinc binding energy of the ZF1. (**F**) Free energy profiles projected onto the conformational space formed by *Q*_ZF2−SL3_ and the zinc binding energy of the ZF2.

Interestingly, although the free zinc fingers are unstable at apo condition as shown in Figs 2 and 6, binding of the RNA makes the ZF1 well folded even without zinc binding (Fig 8A and 8B), which suggests that the target RNA tends to reshape the energy landscape of the zinc-finger domains by interface interactions, leading to increased stability. Such coupled binding and folding has been observed in the target recognition of a number of intrinsically disordered proteins [54, 65–72]. The RNA binding induced stabilization can also be observed for the ZF2, as shown in Fig 8C and 8D. Due to the enhanced stability of the folded state, the zinc affinity has been strengthened significantly upon the RNA binding (Fig 8E and 8F). These results demonstrate the tight interplay between the NCp7 folding, zinc binding, and RNA recognition.

**Fig 9:**
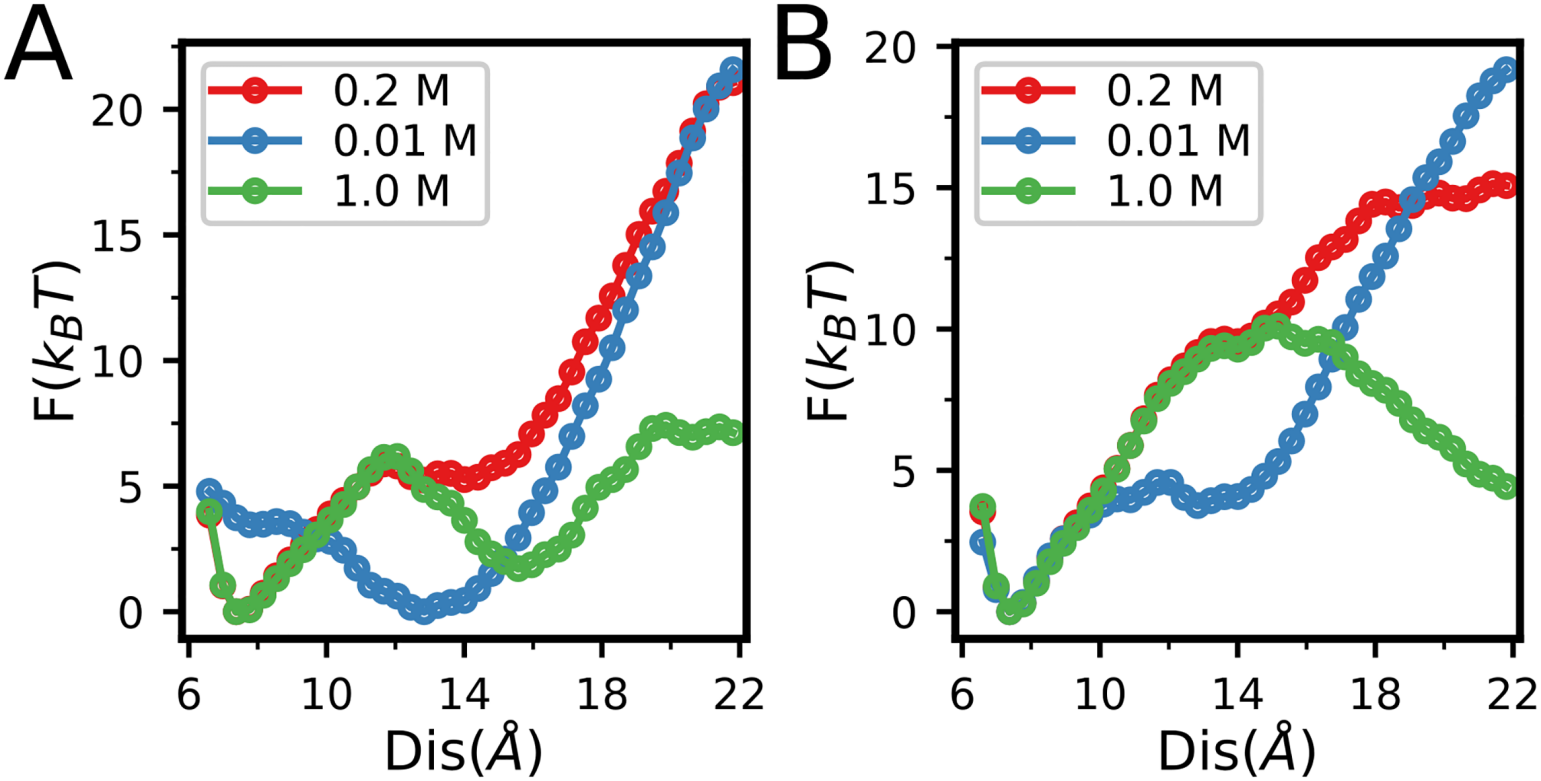
(**A**, **B**) Free energy profiles from umbrella sampling projected onto the distance between the centers of mass of the ZF1 and SL3 at different salt concentrations without Zn^2+^(**A**) and with saturated zinc concentration (**B**).

As the target RNA is highly charged, electrostatic interaction plays important role in the NCp7-RNA recognition process. Therefore, it is interesting to study how electrostatic interaction affect the NCp7 binding to the target RNA. By performing additional umbrella simulations of NCp7 binding to SL3 at low(0.01 M) and high(1.0 M) salt concentrations with and without supplying zinc ions, we showed that the NCp7 achieves optimal balance between the affinity and binding kinetics at the physiologically relevant salt concentration (0.2 M). When the salt concentration is low (0.01 M), the strong electrostatic interaction stabilizes the encounter complex structure, as shown by the low free energy around the distance of 14Å, which tends to slow down the formation of native structure of the complex in Fig 9A. Particularly, at apo condition, the ZF1 tends to be locked at the disordered states, preventing the formation of specific interaction between the ZF1 and RNA (Fig 9B). On the other hand, when the salt concentration is high (1.0 M), the screening effect weakens the electrostatic interactions, reducing the binding affinity between the NCp7 and RNA.

When the electrostatic interaction is screened, cooperative transition is required for ZF1 binding to nuleic acid correctly, especially at the saturated zinc concentration. Previous experimental studies showed that the basic charged residues in the short linker is essential to NCp7 functional dynamics [27]. Furthermore, Wu and coworkers found that mutations on the cationic residues on the short linker suppress the specific stacking of NCp7 to nucleic acids [73]. Taken all these findings together, we speculate that the positively charged residues in the short-linker also have important role in NCp7-nuleic acid recognition process by providing proper electrostatic interaction which facilitates the association of NCp7 to nucleic acid correctly and efficiently.

## Discussion and conclusion

In summary, using a coarse-grained model capable of integratedly describing the ligand binding, protein folding and protein-RNA association, we studied the role of zinc binding on the folding and target RNA recognition of the protein NCp7. Our model well reproduced the differences of stability and zinc binding affinity between the two zinc finger domains observed in previous experimental work. We then showed that the zinc binding not only contributes to the protein stability, but also modulates the structural features of the folding transition state ensemble of the zinc fingers. Zinc binding makes the transition state ensemble less structured, which is in line with the Hammond effect observed typically in mutation, temperature or denaturant induced perturbations to protein structure and stability. The simulations also demonstrated a mutual coupling mechanism in the NCp7-RNA recognition, in which zinc binding enhances the NCp7-RNA affinity, and simultaneously, the binding of RNA tends to reshape the energy landscape of the NCp7, leading to increased protein stability and zinc affinity. Compared to the ZF2, the ZF1 has much larger contribution to the NCp7-RNA affinity mostly arising from the pronounced hydrophobic interactions. As for the ZF2, the zinc ion is essential for its binding to target RNA, since the ZF2 is fully unstructured at apo condition. Zinc binding induces the folding of the ZF2, which further contributes to the specific ZF2-RNA interactions. Due to its low zinc binding affinity and the crucial role in the specific RNA recognition, the ZF2 has often been taken as a potential target in recent anti-NC therapeutic strategies [23, 24, 74, 75]. Further more, our results revealed the important role of the electrostatic interaction during the binding of the NCp7 to the substrate nucleic acids. The NCp7 achieves the optimal balance between the affinity and binding kinetics near the physiologically relevant salt concentrations. Such salt concentration dependence of the NCp7-RNA interactions might be related to the positively charged residues of the short linker. Previous experiment showed that mutations of the cationic residues of NCp7 resulted in nucleic acid interaction defects [73].

In addition, our simulations provided detailed characterizations of the structural features of the inter-domain conformational ensemble. Due to the weak inter-domain interactions between the two zinc fingers, the inter-domain conformational ensemble of the NCp7 is highly heterogeneous and consists of two kinds of conformations with quick equilibrium. In one of the conformations, the inter-domain contacts are well formed, representing a more rigid conformation. Whereas in another conformation, there is no inter-domain contacts, leading to high conformational flexibility. Such heterogeneous distribution of the inter-domain conformational ensemble can be further modulated by zinc binding. Such conformational flexibility may be functionally important, facilitating the the adaptive binding of the NCp7 to different target nuleic acids. Due to the inter-domain interactions, the folding of the two zinc finger domains shows weak cooperativity.

It is worth mentioning that for the sake of computational efficiency, in the current study the effects of zinc ion binding were modelled implicitly by introducing zinc-mediated contacting interactions of the liganding residues. Although such implicit treatment of the ligand binding can reasonably capture the overall effect of zinc binding on the structure and dynamics of the proteins, it cannot provide information on how the zinc ions coordinate to the liganding residues, for which more sophisticated model with explicit considerations of metal ion induced charge transfer, polarization and protonation/deprotonation is needed [60]. In the future, with the rapid increasing of the computational ability, it is interesting to study the coupling between the zinc ion coordination and the structure/dynamics of the NCp7.
